# Bromide of Sodium and Epilepsy

**Published:** 1882-07

**Authors:** 


### BROMIDE OF SODIUM AND EPILEPSY.

Dr. Hammond's experience has proved the following to be one of the best plans of treatment for epilepsy : Dissolve eight ounces of bromide of sodium in a quart of water. Of this take a teaspoonful three times a day. After three months add one teaspoonful more to the night dose, and after another three or four months add a teaspoonful to the afternoon dose also. At the expiration of a year do the same with the morning dose, and continue with this for a year or more thereafter. If no symptoms of the disease have meanwhile appeared, then gradually reduce the doses, and at the expiration of the third year stop. The attacks do not usually return after this course of treatment. Ordinarily, however, patients stop the medicine after a month or two, and in such cases the attacks almost invariably return. It is then almost impossible to bring these patients under the influence of the bromides again. The doses will have to be at least doubled, and this may so derange the system as to make it impossible to take the medicine longer.—*Cincinnati Med. News.*

